# Delayed aortic injury following multiple rib fractures: a case report

**DOI:** 10.1186/s44215-025-00225-2

**Published:** 2025-10-14

**Authors:** Kei Nakano, Tomohiko Matsuzaki, Shota Fujino, Masaya Ohara, Takashi Ishihara, Kazuhiro Matsuo, Tomoki Higeta, Kie Maita, Hiroto Onozawa, Takaaki Tsuboi, Atsushi Wada, Naohiro Aruga, Tomoki Nakagawa, Ryota Masuda

**Affiliations:** Department of General Thoracic Surgery, Department of Surgery, Tokai University School of Medicine, 143 Shimokasuya, Isehara, Kanagawa 259-1193 Japan

**Keywords:** Delayed hemothorax, Blunt thoracic trauma, Traumatic aortic injury

## Abstract

**Background:**

Delayed hemothorax due to aortic injury is rare, and its delayed diagnosis can be fatal. Herein, we report a case of delayed hemothorax owing to aortic injury following blunt chest trauma, which was successfully treated through emergency surgery.

**Case presentation:**

A woman in her 70s fell on an escalator at a train station and was brought to our emergency department. Initial evaluation with computed tomography (CT) imaging revealed a thoracic vertebral fracture, bilateral lower rib fractures, and a right-sided hemothorax. She was admitted for observation and supportive care, and her condition remained stable for days. On day 9 of hospitalization, the patient suddenly experienced cardiac arrest. A plain chest X-ray showed a massive pleural effusion on the left side, prompting the insertion of a chest tube. The drainage was hemorrhagic, and laboratory tests showed a significant decline in the hemoglobin level to 4.9 g/dL. Following successful cardiopulmonary resuscitation and the return of spontaneous circulation, a contrast-enhanced CT scan of the chest was performed. However, no active extravasation or apparent source of bleeding was observed. Owing to ongoing hemodynamic instability and the substantial volume of hemothorax, emergency surgery was conducted to identify and control the source of hemorrhage. Intraoperatively, after evacuating a large hematoma from the left thoracic cavity, a small ulcerated hole was identified in the descending thoracic aorta, which appeared tortuous. Pulsatile bleeding was observed from this site. Manual compression was applied to achieve temporary homeostasis. Intraoperative ultrasound showed no evidence of aortic dissection. The bleeding site was sutured directly, and hemostasis was achieved. No additional significant intrathoracic injuries were identified. The aortic injury had resulted from mechanical irritation or penetration by the adjacent fractured lower rib. This represented a case of delayed hemothorax secondary to traumatic aortic injury, a rare but potentially fatal complication.

**Conclusions:**

In cases of delayed hemothorax following blunt thoracic trauma—particularly with fractures of the lower left posterior ribs, clinicians should maintain a high index of suspicion for aortic injury. Prompt recognition and surgical intervention are critical for patient survival in such cases.

## Background

Delayed hemothorax associated with chest trauma is rare and commonly attributed to intercostal artery injury resulting from rib fractures. Conversely, delayed hemothorax due to aortic injury is rare, and delayed diagnosis can be fatal if not promptly diagnosed. We report a case of delayed hemothorax owing to aortic injury following blunt thoracic trauma that was successfully treated with emergency surgery.

### Case presentation

This case involved a woman in her 70s who presented with chest and back pain following a fall on an escalator at a train station during nighttime hours. She had a medical history of hypertension, hyperlipidemia, and hiatal hernia. The patient initially sought medical attention at another hospital. She was diagnosed with a thoracic vertebral fracture, multiple left rib fractures, and a right-sided hemothorax. The patient was subsequently transferred to our hospital for further evaluation and management.

### Findings at admission

**Fig. 1:**
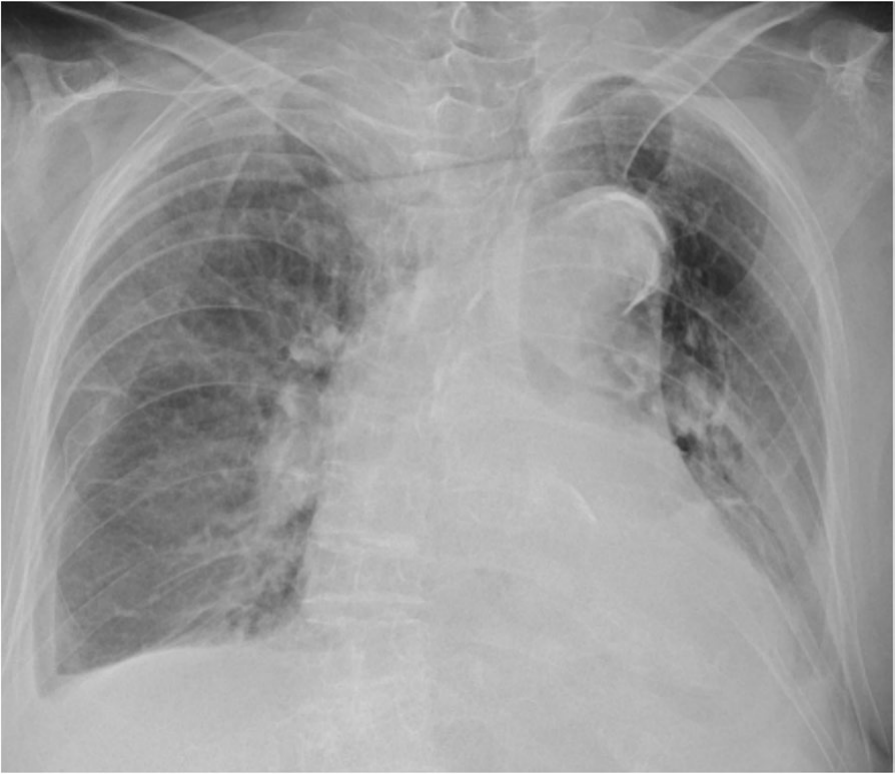
Chest X-ray image showing a mild right-sided pleural effusion

**Fig. 2:**
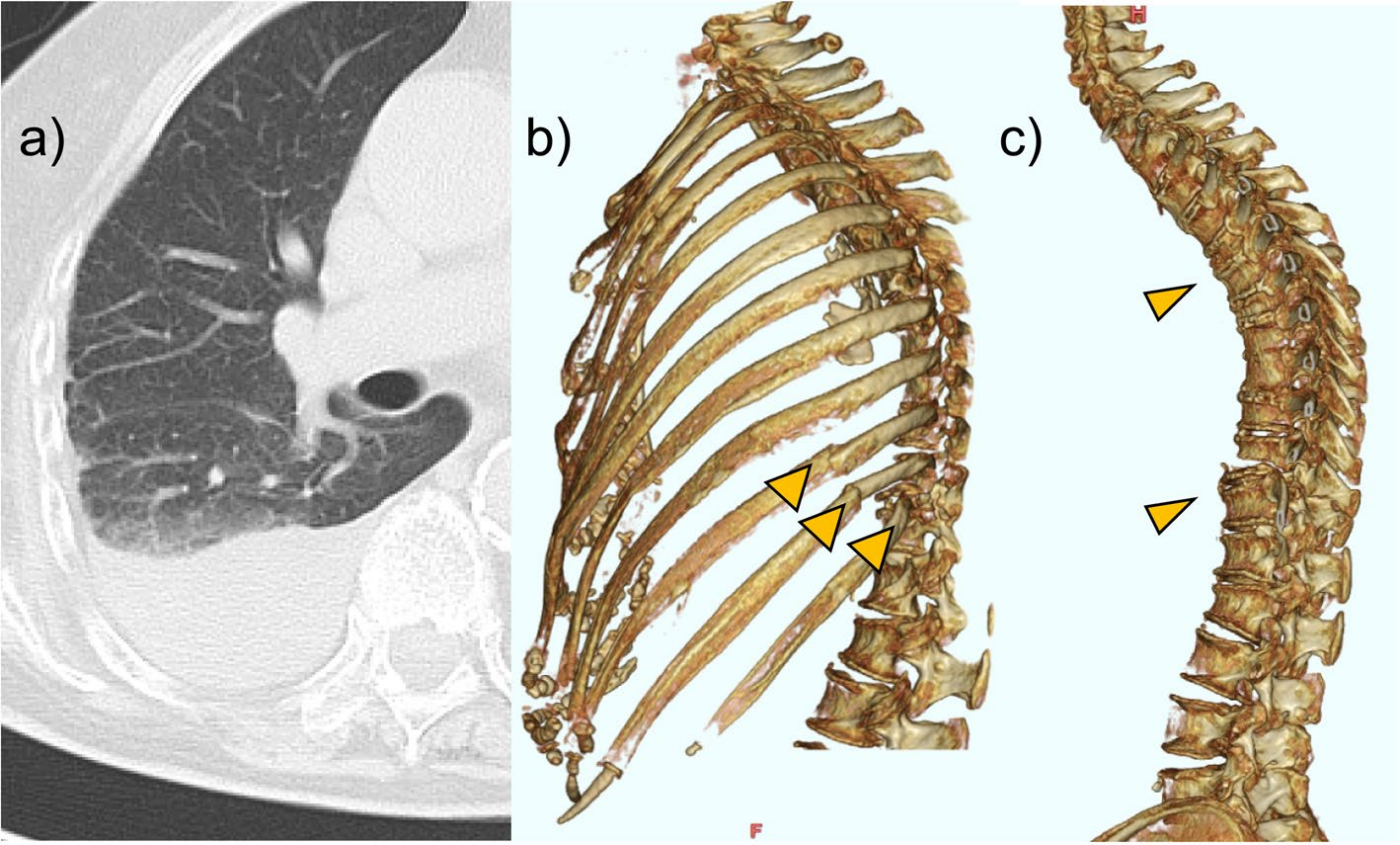
CT images obtained during transport. **a** Right hemothorax, **b** fractures of the 9th–11th ribs on the left side, and **c** multiple thoracic vertebral fractures. CT, computed tomography

The laboratory findings of the patient on arrival were as follows: Japan Coma Scale, 0; respiratory rate, 24/min; heart rate, 80/min; blood pressure, 144/96 mmHg; body temperature, 36.2 °C; and SpO_2_, 95% while receiving 2 L/min of oxygen via nasal cannula. Initial blood test results were as follows: white blood cell count, 13.5 × 10^3^/μL; red blood cell count, 330 × 10^4^/μL; hemoglobin (Hb) level, 10.4 g/dL; platelet count, 16.4 × 10^5^/μL; aspartate aminotransferase level, 39 U/L; alanine transaminase level, 2 U/L; lactate dehydrogenase level, 609 U/L; and C-reactive protein level, 10.6 mg/dL. A plain chest X-ray at admission revealed a right-sided pleural effusion (Fig. 1). CT imaging showed fractures of the left 9th–11th ribs, multiple thoracic vertebral fractures, and a right-sided hemothorax. The displacements of the fractured ribs were mild (Fig. 2).

### Hospital course

**Fig. 3:**
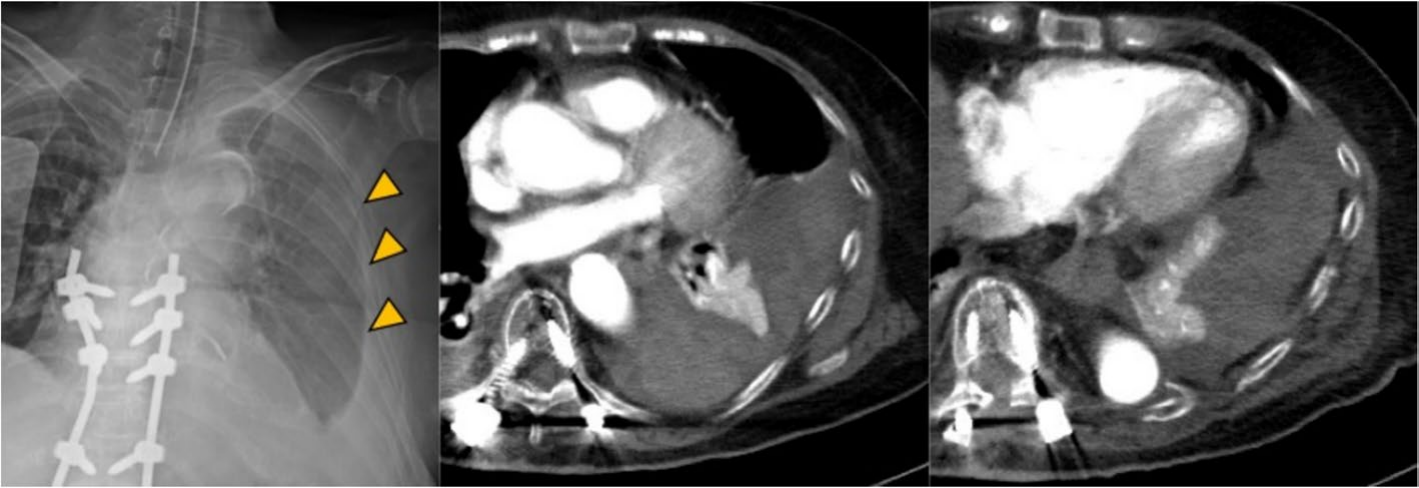
Imaging findings at the time of cardiac arrest reveal a worsening of the left hemothorax, while no obvious source of bleeding was identified

Because the displacement of the left rib fracture was mild, a conservative observation policy was adopted. A chest tube was inserted to manage the right-sided hemothorax. Surgical fixation of the multiple thoracic vertebral fractures was subsequently performed at the orthopedic department. Postoperative rehabilitation, including the initiation of ambulation, proceeded smoothly, and the chest tube was removed on day 7 post-injury. On the morning of day 9, as the patient changed her position to get out of bed, she suddenly developed dyspnea and chest pain, which was followed by cardiac arrest. Laboratory tests revealed significant anemia (Hb level 4.9), and increased left pleural effusion was noted (Fig. 3). Bloody drainage was observed after the chest tube reinsertion, totaling 300 mL, after which only a minimal amount of bloody drainage was observed. After six cycles of cardiopulmonary resuscitation, spontaneous circulation was restored. Subsequent contrast-enhanced chest CT revealed no obvious bleeding source (Fig. 3). The patient was referred to our department for further investigation of the hemorrhage. A fracture of the left lower rib was identified, and delayed hemothorax owing to intercostal arterial or diaphragmatic injury was considered, thereby leading to the decision to perform emergency surgery.

### Intraoperative findings

**Fig. 4:**
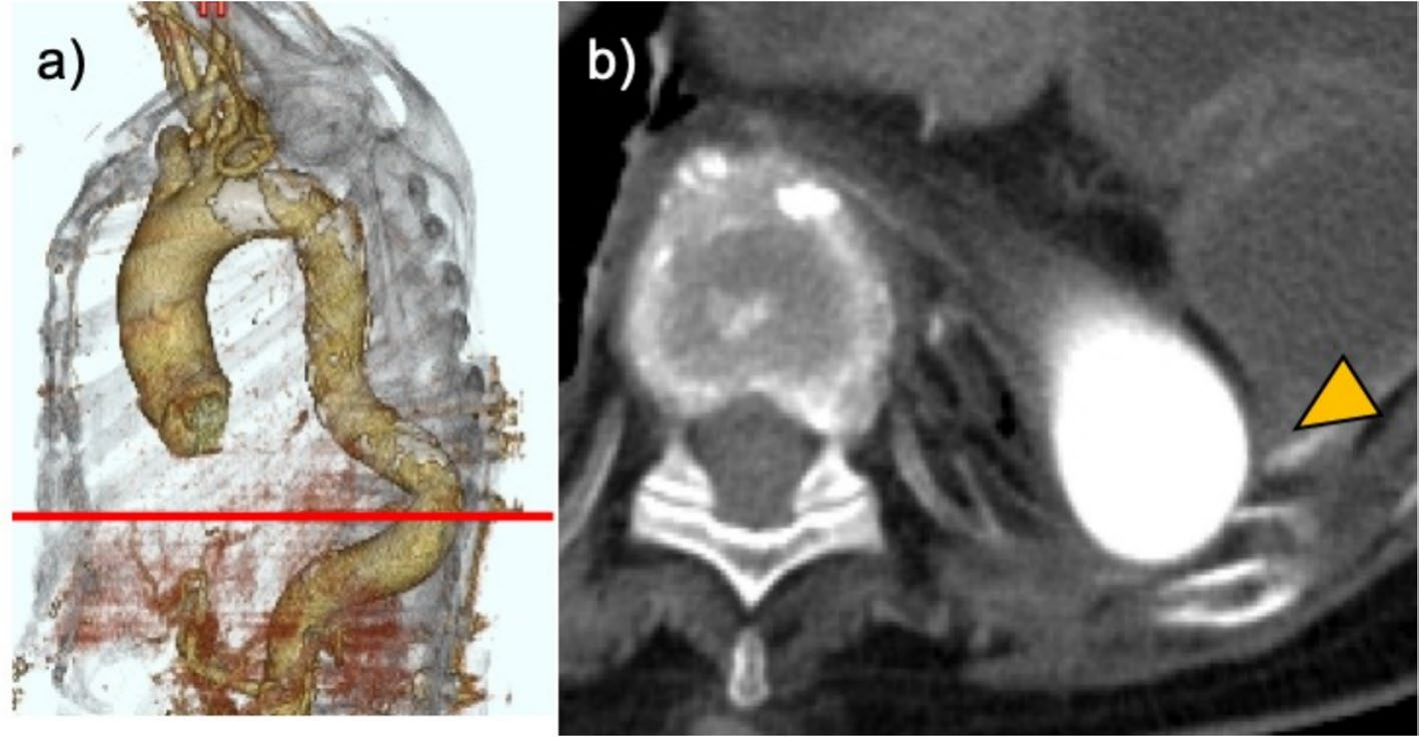
CT image of the injured area: **a** aortic tortuosity. **b** Displacement of the fractured end of the left 10th rib, which is in close proximity to the tortuous part of the aorta. CT, computed tomography

**Fig. 5:**
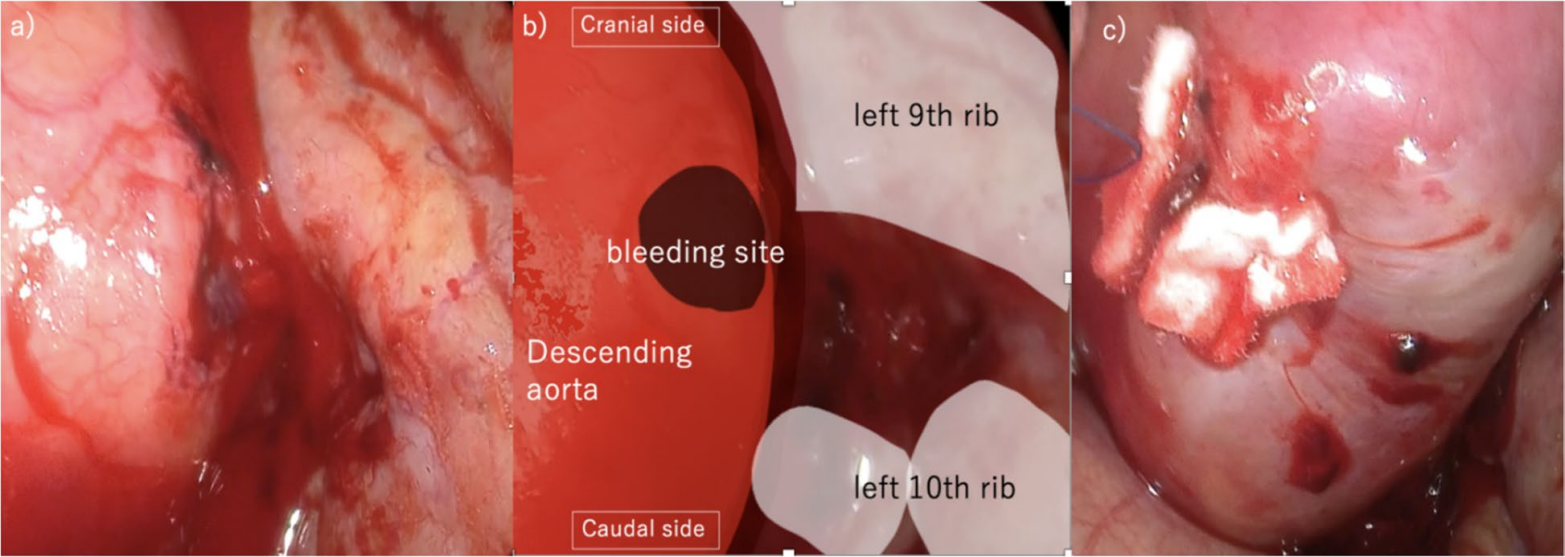
Intraoperative findings. **a** Ulcerative changes were observed in the descending aorta, with pulsatile bleeding originating from that area. **b** Schematic illustration showing the anatomical relationships. **c** Suture repair

An initial attempt at thoracoscopic examination was made by creating an access port at the left fifth intercostal space. However, upon entry, active outflow of bloody fluid was encountered, prompting an immediate switch to a posterolateral thoracotomy. The thoracic cavity was filled with blood and clots. After suctioning the intrathoracic blood and removing the blood from the surface of the descending aorta, the 10th rib was in close proximity to the tortuous portion of the aorta, suggesting that this was the injury site (Fig. 4). While no sharp protrusion of the rib fracture site into the thoracoscopic field was observed, ulcerative alterations in the descending aorta were observed at the site of rib compression, and pulsatile bleeding was observed from that site (Fig. 5). The bleeding point was immediately compressed, achieving temporary hemostasis. Given the possibility of dissection at the descending aorta injury site, intraoperative ultrasonography was performed in the presence of a cardiovascular surgeon; no signs of dissection were confirmed. The bleeding site was sutured with a 4-0 Prolene suture by using a 26-mm Pre-Jet needle, and complete hemostasis was achieved. During surgery, no sharp edges were observed at the fracture site of the rib adjacent to the aorta, and that site was left untreated. A 32Fr Silastic drain was inserted, and the surgery was completed. The operative time was 111 min, and the total blood loss was 1992 mL (Fig. 5).

### Postoperative course

We planned to perform prosthetic graft replacement if a new dissection was detected after the operation. However, circulatory stability was promptly achieved following hemostasis. The level of consciousness of the patient improved postoperatively, and she was subsequently transferred for rehabilitation. A follow-up CT scan performed 4 months afterwards revealed neither dissection nor aneurysm formation.

### Discussion and conclusions

Delayed hemothorax does not have a universally accepted definition. However, many reports describe it as a hemothorax that develops or worsens several hours after an injury. Hemothorax occurs in approximately 37% of patients with blunt chest trauma [1], while delayed hemothorax occurs in approximately 4–7% of cases [2, 3]. The most common cause is intercostal artery injury from rib fractures [4]. Other causes include injuries to the diaphragm [5], azygos vein [6], and, as in this case, aorta [7].

Reports of delayed hemothorax from thoracic aortic injury are rare [8]. In all reported cases, the injury was associated with fractures of the lower left ribs [8, 9]. In our case, fractures of the left 9th–11th ribs were observed, with the posterior edge of the 10th rib displaced near the tortuous descending aorta, suggesting this as the injury site. Because no circulatory changes occurred for several days after the injury, we presumed that the rib edge shifted and injured the aorta when the patient changed position on the morning of cardiac arrest. Therefore, when posterior lower rib fractures lie close to the descending aorta, aortic injury should be suspected and the patient closely monitored.

In this case, the delayed hemothorax developed on day 9 post-injury. Previous reports describe onset between 2 and 15 days after injury [8], with some cases occurring during patient repositioning, as seen in this case [10]. In contrast, 80% of patients with aortic injury reportedly die before reaching the hospital [11], and some may die suddenly without diagnosis. Therefore, the optimal duration of strict monitoring remains unclear. This patient happened to be hospitalized for orthopedic rehabilitation when the delayed bleeding from the aorta occurred, which allowed for timely resuscitation and surgical intervention. Had this event occurred at home, survival would have been unlikely. In particular, for patients with posterior lower rib fractures near the descending aorta, hospitalization until stable ambulation is achieved may be warranted, even in the absence of symptoms. Emergency or semi-emergency surgery, including fixation of rib fractures, removal of rib bone fragments, and video-assisted thoracoscopic surgery for intrathoracic observation—screening for injuries such as those of the diaphragm or pericardium—should be considered in patients with lower rib fractures, owing to the risk of penetrating cardiac injury in the case of anterior rib fractures and injury to the descending thoracic aorta in the case of posterior rib fractures [12], as in this case. More case reports are needed to guide clinical decisions.

Few reports have successfully documented aortic repair using direct suture closure [8]. If dissection is present, suturing may worsen the injury. In our case, intraoperative ultrasound examination was performed with cardiovascular surgeons to confirm the absence of dissection, allowing direct suture repair. Prompt management of aortic injury is essential for survival. Our patient experienced cardiac arrest during repositioning, but rapid hemostasis saved her life.

Surgical intervention for hemothorax is generally indicated when: (i) initial chest tube output exceeds 500 mL; (ii) drainage exceeds 1500 mL within the first hour; (iii) continuous bleeding of >200 mL/h persists for 2–4 h; or (iv) continuous transfusions are required to maintain hemodynamic stability [13, 14]. If any of these conditions are met, the drain should be clamped immediately, and emergency surgery should be conducted. However, in cases similar to ours—where cardiac arrest occurs despite minimal chest tube output—caution is advised. In aortic injury, massive bleeding and hypotension usually occur immediately after injury, but initial drainage may be limited by clot formation. Therefore, even if the criteria are not met, surgical intervention should be guided based on vital signs and imaging. When sudden cardiac arrest or rapid hypotension occurs with suspected intrathoracic bleeding, decisions should not rely solely on chest tube output; prompt hemostatic surgery is essential.

Our case of delayed aortic injury following multiple chest trauma should alert physicians to the possibility of descending aortic injury in the case of sudden hypotension, cardiac arrest, or worsening hemothorax. Surgical decisions should be guided based on a comprehensive evaluation, not just drain output, and hemostasis should be promptly achieved.

## Data Availability

All data generated or analyzed during this study are included in this article. Further information is available from the corresponding author on reasonable request.

## Abbreviations

CT: Computed tomography
Hb: Hemoglobin
